# Crosstalk between mucosal microbiota, host gene expression, and sociomedical factors in the progression of colorectal cancer

**DOI:** 10.1038/s41598-022-17823-7

**Published:** 2022-08-04

**Authors:** Namjoo Kim, Jeong-An Gim, Beom Jae Lee, Byung il Choi, Hee Sook Yoon, Seung Han Kim, Moon Kyung Joo, Jong-Jae Park, Chungyeul Kim

**Affiliations:** 1 Division of Gastroenterology, Department of Internal Medicine, Korea University Guro Hospital, Gurodong-gil 97, Guro-gu, Seoul, 152-703 Republic of Korea; 2 Medical Science Research Center, College of Medicine, Korea University Guro Hospital, Seoul, Republic of Korea; 3 Department of Pathology, College of Medicine, Korea University, Seoul, Republic of Korea

**Keywords:** Computational biology and bioinformatics, Genetics, Microbiology, Gastroenterology

## Abstract

Various omics-based biomarkers related to the occurrence, progression, and prognosis of colorectal cancer (CRC) have been identified. In this study, we attempted to identify gut microbiome-based biomarkers and detect their association with host gene expression in the initiation and progression of CRC by integrating analysis of the gut mucosal metagenome, RNA sequencing, and sociomedical factors. We performed metagenome and RNA sequencing on colonic mucosa samples from 13 patients with advanced CRC (ACRC), 10 patients with high-risk adenoma (HRA), and 7 normal control (NC) individuals. All participants completed a questionnaire on sociomedical factors. The interaction and correlation between changes in the microbiome and gene expression were assessed using bioinformatic analysis. When comparing HRA and NC samples, which can be considered to represent the process of tumor initiation, 28 genes and five microbiome species were analyzed with correlation plots. When comparing ACRC and HRA samples, which can be considered to represent the progression of CRC, seven bacterial species and 21 genes were analyzed. When comparing ACRC and NC samples, 16 genes and five bacterial species were analyzed, and four correlation plots were generated. A network visualizing the relationship between bacterial and host gene expression in the initiation and progression of CRC indicated that *Clostridium spiroforme* and *Tyzzerella nexilis* were hub bacteria in the development and progression of CRC. Our study revealed the interactions of and correlation between the colonic mucosal microbiome and host gene expression to identify potential roles of the microbiome in the initiation and progression of CRC. Our results provide gut microbiome-based biomarkers that may be potential diagnostic markers and therapeutic targets in patients with CRC.

## Introduction

Colorectal cancer (CRC) is one of the most common carcinomas worldwide^1,2^. Despite CRC screening programs, including fecal immunochemical tests and colonoscopy in worldwide, CRC still has a high incidence and mortality^3,4^. Multiple studies have shown that the gut microbiome is a crucial environmental factor that can regulate human health, and genomic changes in the gut microbiota can contribute to a variety of human diseases, including malignant disease, chronic inflammatory diseases, and metabolic disease^5–10^. The gut microbiota plays an important role in the regulation of gut homeostasis. It can metabolize the indigestible components of food, synthesize nutrients for epithelial regeneration, and modulate the immune response to maintain mucosal integrity by protecting against harmful environmental and endogenous toxic stimuli^11–14^. The initiation and progression of CRC are related to complex biological pathways involving multiple genetic and epigenetic alterations^15–17^. Many reports have shown that dysbiosis is closely associated with the initiation and progression of CRC and that the gut microbiome can be a candidate marker for early detection of CRC^18–25^. Therefore, modulation of the gut microbiome has been attempted as an adjunctive therapeutic strategy for CRC, such as increasing the sensitivity of immune checkpoint inhibitors in advanced and metastatic CRC^26–28^. With the development of bioinformatics analysis, accumulated omics data have been widely used to investigate the pathogenesis of CRC^29,30^. Multi-omics data are also rapidly expanding knowledge of metagenomic and host gene expression in health and disease^31^.

Some bacterial taxonomic groups have been found to be significantly correlated with the methylation or demethylation of host genes. However, the role of gut microbes as environmental factors in the initiation and progression of CRC and the interaction between microbes and host genes during CRC tumorigenesis remain unclear and need to be elucidated. Previously, we assessed the differentially expressed genes (DEGs) among high-risk adenoma (HRA), advanced CRC (ACRC), and normal control (NC) samples using RNA sequencing (RNA-seq) to identify candidate genes that play a role in CRC progression^32^. In this study, we integrated the results of simultaneous metagenomic sequencing and RNA-seq on the same colonic mucosa in HRA, ACRC, and NC samples to analyze the correlation between bacterial species and host gene expression and define the role of gut microbiota-host gene crosstalk in CRC development and progression. To validate and exclude the interference of environmental factors on metagenomics and gene expression, sociomedical factors such as dietary patterns, socioeconomic status, medical/family history, and psychiatric factors were included in this integrated analysis^33,34^.

## Results

### Integrated patterns of HRA, ACRC, and NC samples

We retrieved gene expression data, microbiome data, and survey results from RNA-seq, metagenome analysis, and a sociomedical questionnaire, respectively. The RNA-seq data included 46,851 features, and 763 features were selected based on significant differences between the three groups (analysis of variance [ANOVA]; p-value < 1 × 10^–6^). The metagenome analysis yielded 529 features, each of which corresponded to a microbe at the species level from 16S rRNA sequencing data. Ninety-three continuous variables were selected from the survey results. Each of the three datasets was normalized to a value between 0 and 1, and the three datasets were merged into one table (30 samples with 1385 features).

**Figure 1:**
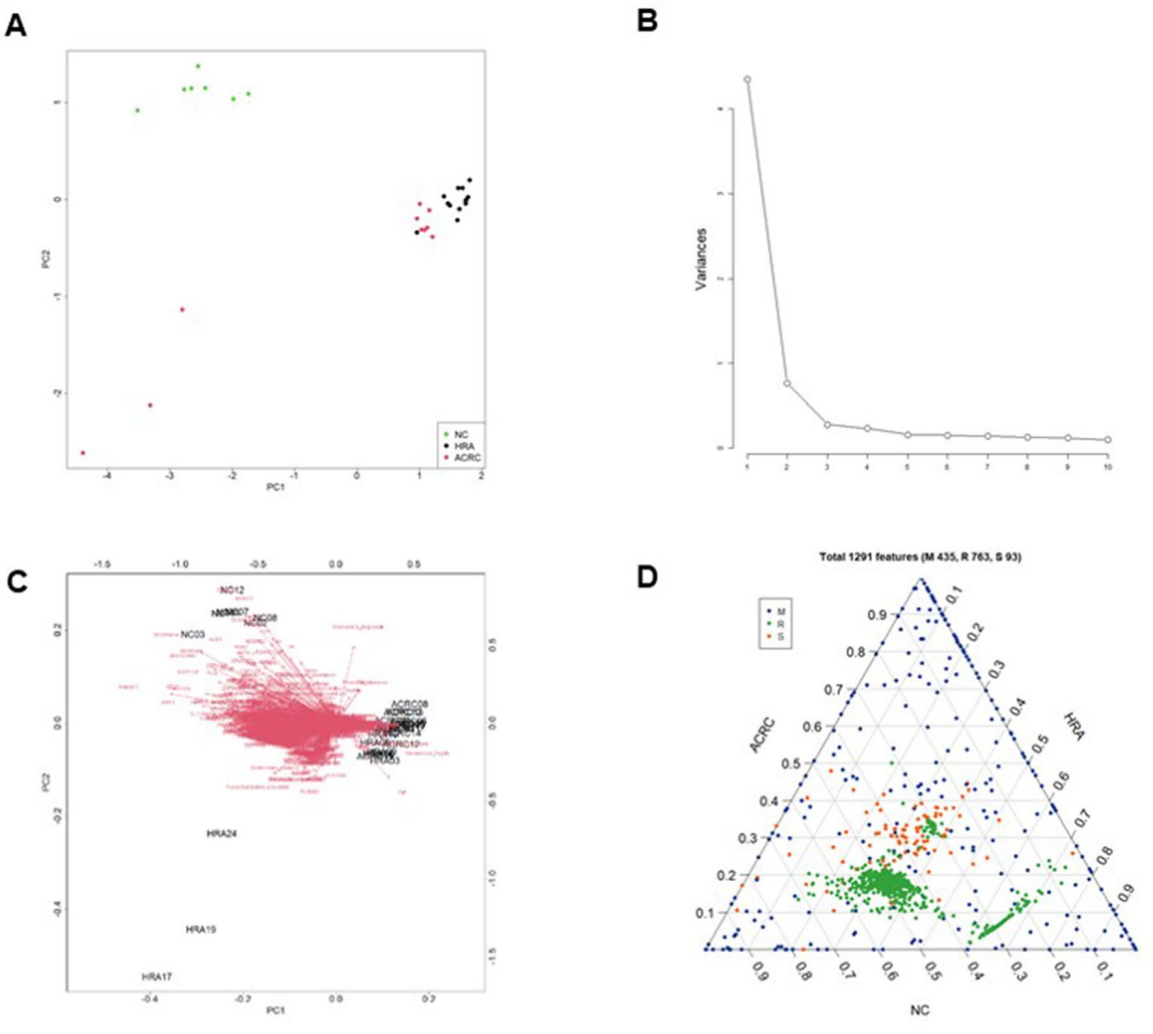
Integrative analysis and visualization of the 30 samples using three datasets. In the 30 samples (13 advanced colorectal cancers [ACRCs], 10 high risk adenomas [HRAs], and 7 normal controls [NCs]), there were 529 species identified by metagenome analysis and 763 genes identified by RNA sequencing. The survey results identified 93 associated variables. Each dataset was merged and normalized to a value between 0 and 1. Principal component analysis (PCA) was applied to reduce the dimension of features from each dataset, and 10 principal components (PCs) were retrieved. (**A**) Each two-dimensional PCA plot represents two PCs, reduced from 1385 features. (**B**) The scree plot represents 10 PCs. (**C**) The biplot represents the direction of each feature. (**D**) In the ternary plot, 1385 features corresponding to ACRCs, HRAs, and NCs are presented as points and are distinguished by three colors.

We used PCA as a dimension reduction tool to observe the merged datasets of gene expression data, microbiome data, and sociomedical patterns of the survey results. The matrix separated the ACRC, HRA, and NC groups. The merged multi-omics data consisted of 30 samples (Fig. S1 and Table S1), and a total of 1385 features were diminished to 10 principal components (PCs). The first and second PCs are listed in the PCA plot (Fig. 1A). In the PCA plot, a two-dimensional plot represents two components, reduced from the 1385 merged and normalized matrices. Each plot indicates a sample, and the three groups are plotted in different colors (Fig. 1A). The variances of each PC were graphed as a scree plot. The top four components accounted for 78.14% of the variance, and the top two components accounted for 71.13% of the variance in the total dataset. In the scree plot, the x-axis indicates the top 10 PCs, and the y-axis represents the variance of each PC captured from a total of 1385 features (Fig. 1B). By merging the PCA plot and vectors from the loadings of variables, a biplot was created (Fig. 1C). The ternary plot shows the relative abundance of the 1385 features. The three sides of the triangle represent the relative abundance of the three groups. As the two sides approach the vertex where they meet, the relative abundance between the two pairs is different (Fig. 1D). These results show a clear clustering of metagenomic, RNA-seq, and sociomedical factors associated with the tumors and the NC samples, except for three HRA samples.

### Abundance and diversity of the colonic mucosal microbiome in HRA and ACRC samples

**Figure 2:**
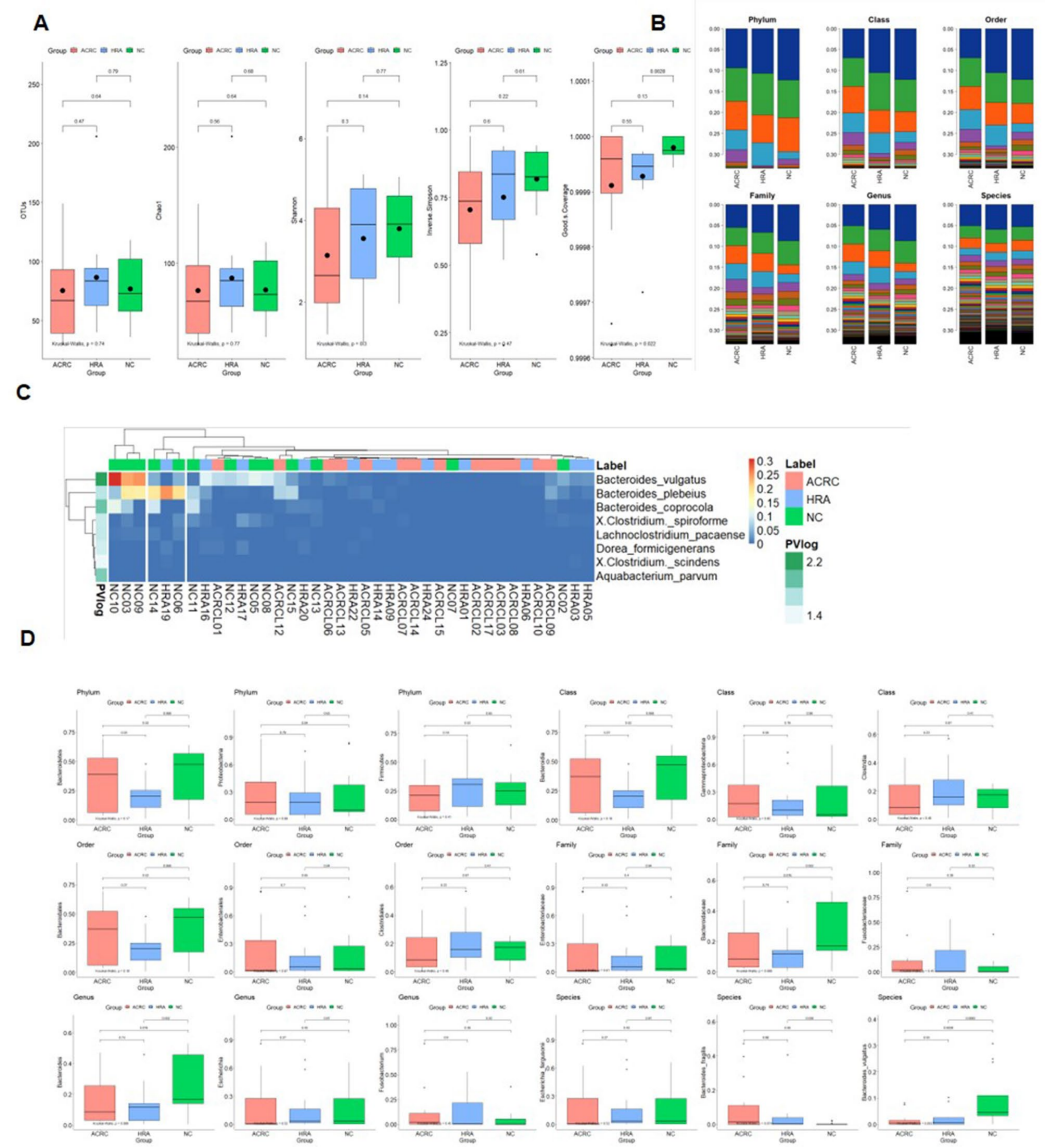
Alpha diversity, distribution, and different patterns of the metagenome analysis. (**A**) Five diversity indices were visualized as boxplots. Points and horizon bars indicate the means and the medians of each group, respectively. The operational taxonomic unit (OTU), the Chao1, the Shannon index, the inverse Simpson index, and the Good’s coverage are provided. The three groups are advanced colorectal cancer (ACRC; n = 13), high-risk adenoma (HRA; n = 10), and normal controls (NC; n = 7). (**B**) Relative abundance of each classification level. The relative abundance of each sample is listed by phylum, class, order, family, genus, and species. (**C**) Heatmap showing the relative abundance of significantly different bacterial species between the three groups. Of 528 species, 8 were significantly different between the three groups (ANOVA, *p* < 0.05). Each row indicates 8 species and is classified by group. Statistical significance is indicated as a row annotation bar, and darker green indicates greater significance. Each column represents an individual patient, and their labels are indicated as a column annotation bar. Each cell of the heatmap indicates the relative abundance of species, with colors gradually changing from blue to red, corresponding to low and high relative abundance, respectively. (**D**) Boxplots showing the ratio of the abundance in each group of the top three taxa in six classification levels. In each boxplot analysis, the statistical significance of the three pairings (ACRC vs. NC, ACRC vs. HRA, and HRA vs. NC) was analyzed by *t*-test, the overall significance was analyzed by Kruskal–Wallis test, and the *p*-value is presented. *ANOVA* analysis of variance.

We tried to identify the interactions between the microbiome and host cells in the colonic mucosa of the three groups (Fig. 2A). We estimated the diversity and richness of the microbial communities in mucosal biopsies. Microbiome diversity and richness were obtained as indices at the operational taxonomic unit (OTU) level. Species diversity was determined using the Shannon and Simpson indices. Species richness was defined as the observed number of species assigned to the OTUs detected in each sample. Richness was retrieved from the observed number of species using Chao1. On average, 75.36, 86.50, and 76.92 OTUs were detected in the ACRC, HRA, and NC samples, respectively. The average Chao1 values were 76.49 in ACRC samples, 77.12 in NC samples, and 87.33 in HRA samples. One patient each with HRA and NC had abnormally high OTUs, and no statistically significant difference in diversity was found between the groups.

Decreasing patterns in the three indices (Shannon, Inverse Simpson, and Good’s Coverage) were observed for disease samples compared to NC samples, but there was no significant difference in expression. In the diversity analysis, different patterns were observed between the three groups; however, only Good’s coverage was significantly different. We further compared the microbiome abundances between the three groups at six taxonomic levels (phylum, class, order, family, genus, and species) (Fig. 2B). Taken together, there was no difference in the abundance and diversity of microbiota taxa between HRA, ACRC, and NC samples.

### Functional analysis and detection of bacterial species in HRA and ACRC samples

We attempted to detect and identify the colonic mucosa bacteria associated with CRC development and progression. The significantly associated eight species were selected based on the results of the ANOVA comparing the three groups (Fig. 2C and Table S2; *p* < 0.05). Each row in the heatmap indicates a species identified by the ANOVA results, and each column indicates a sample. The column annotation bar indicates three classes of samples. Each row and column pair is clustered using *k*-means clustering. Each column was split into three groups, and the NC group was clustered. Three species of *Bacteroides* were detected at higher levels in the NC samples. At each classification level, the top three taxa were compared among the three groups. Taxons belonging to the same lower level were included for frequently detected taxa. Therefore, values showing a similar pattern were observed at each classification level. Although no statistically significant differences in abundance were found between the three groups at all classification levels, we could retrieve eight species correlated with the initiation and progression of CRC. *Bacteroides fragilis* had significantly different abundances in HRA and NC samples, and *Bacteroides vulgatus* had significantly different abundances in ACRC and NC samples and in HRA and NC samples. The Kruskal–Wallis test results between the three groups were statistically significant (*p* = 0.0037) (Fig. 2D).

### Predicted function of bacterial species correlated with CRC-associated gene expression

**Figure 3:**
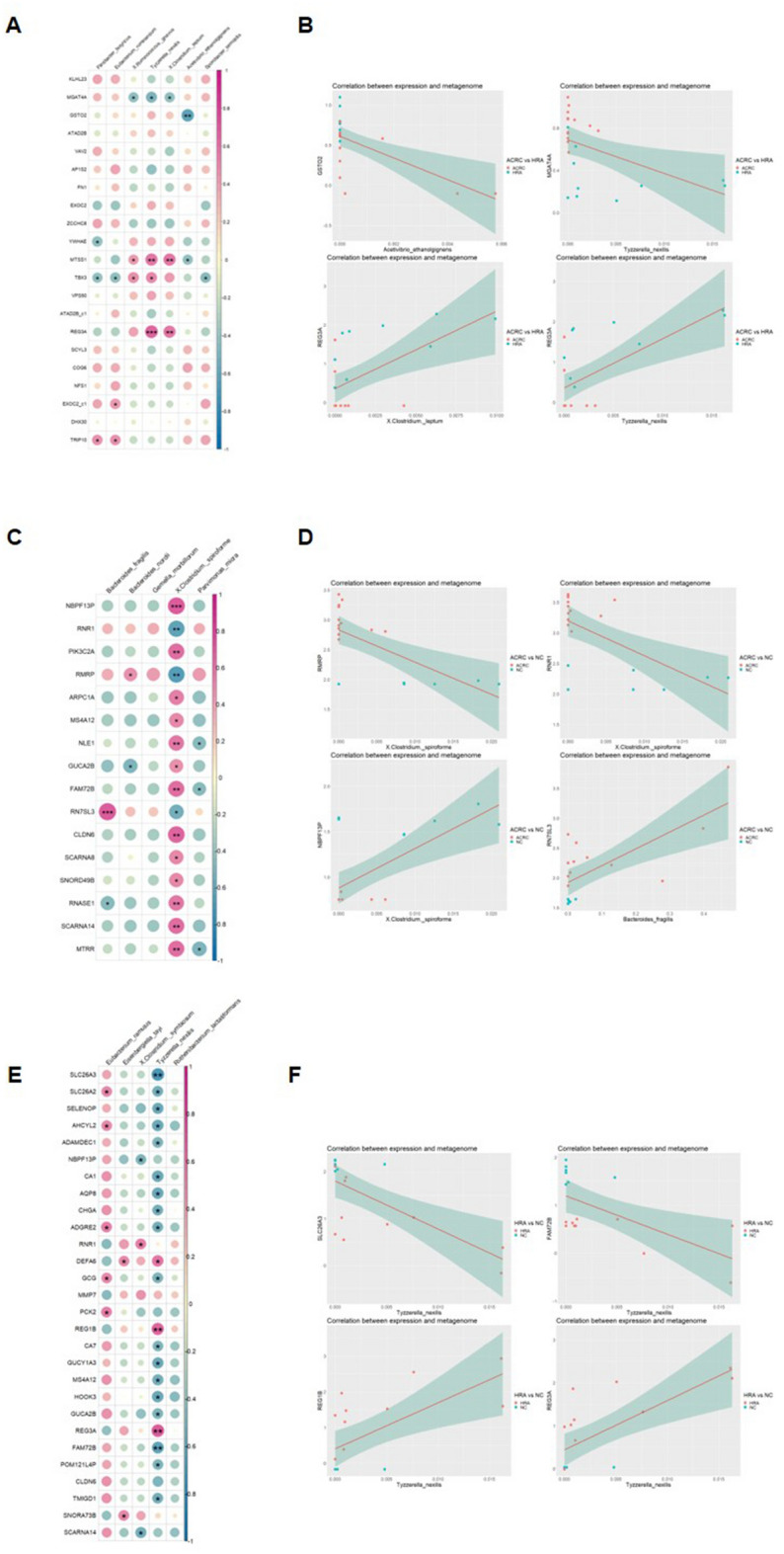
Correlation between RNA sequencing and metagenome results. (**A**) Correlation plots and (**B**) scatter plots comparing ACRC and HRA samples. (**C**) Correlation plots and (**D**) scatter plots comparing ACRC and NC samples. (**E**) Correlation plots and (**F**) scatter plots comparing HRA and NC samples. In correlation plots, color indicates correlation coefficients, and circle size indicates statistical significances. Only correlation coefficients with p-values < 0.05 are shown. The two analysis results with the highest positive and negative correlation coefficients are presented as scatter plots and regression lines. *p*-values of each correlation analysis are indicated in the correlation plots (*p < 0.05, **p < 0.01, ***p < 0.001). *ACRC* advanced colorectal cancer, *HRA* high-risk adenoma, *NC* normal control.

To investigate the role of mucosal bacteria in CRC initiation and progression, we assessed the correlation between gene expression and microbial distribution in the three groups. The initiation and progression of CRC are related to complex biological pathways involving multiple genetic and epigenetic alterations^16,19^. HRA is known to be the precursor of CRC, and the adenoma-carcinoma sequence is the classic mechanism of the development of ACRC. We selected the DEGs in three pairings: ACRC versus HRA samples to represent CRC progression (Fig. 3A,B), HRA versus NC samples to represent CRC initiation (Fig. 3C,D), and ACRC versus NC samples (Fig. 3E,F). Each bacterium and gene was selected by fold change (FC) and p-value (PV) in the *t*-test. We visualized all correlations between species abundance and host gene expression (Fig. 3). In all three pairings, each correlation was provided as a correlation plot and scatter plots. In the correlation plot, relationships of species abundance and host gene expression with PV < 0.05 are indicated as plots, and the plot is red if the correlation is negative.

In the ACRC-HRA pairing, 21 genes (PV < 0.001 and |FC| > 0.4) and seven bacterial species were examined (Fig. 3A). The top two positively and negatively correlated genes and microbiome species are provided as correlation plots (Fig. 3B).

In the ACRC-NC pairing, 16 genes (PV < 0.001 and |FC| > 0.75) and five microbiome species were analyzed, and four correlation plots were created (Fig. 3C,D). All 16 genes were significantly correlated with *Clostridium spiroforme*. *C. spiroforme* had low abundance. *RMRP* and *RNR1* were more highly expressed in ACRC samples than in NC samples, and *NBPF13P* was expressed at lower levels in ACRC samples. The correlation coefficients of *C. spiroforme* with *RMRP*, *RNR1*, and *NBPF13P* were − 0.644, − 0.636, and 0.631, respectively.

In the HRA-NC pairing, 28 genes (PV < 0.001 and |FC| > 0.75) and five microbiome species were analyzed using correlation plots (Fig. 3E,F). Twenty-two genes were significantly correlated with *T. nexilis;* 19 and 3 genes showed negative and positive correlation patterns, respectively. The correlation plots showed four genes that were correlated with *T. nexilis: SLC26A3*, *FAM72B*, *REG1B*, and *REG3A*. We selected four species-gene pairs using the two top and bottom correlation coefficients. The correlation coefficients were − 0.605, − 0.490, 0.589, and 0.646 for the *GSTO2* and *Acetivibrio ethanolgignens* pair, the *MGAT4A* and *T. nexilis* pair, the *REG3A* and *Clostridium leptum* pair, and the *REG3A* and *T. nexilis* pair, respectively.

### Network and visualization of multi-omics data

**Figure 4:**
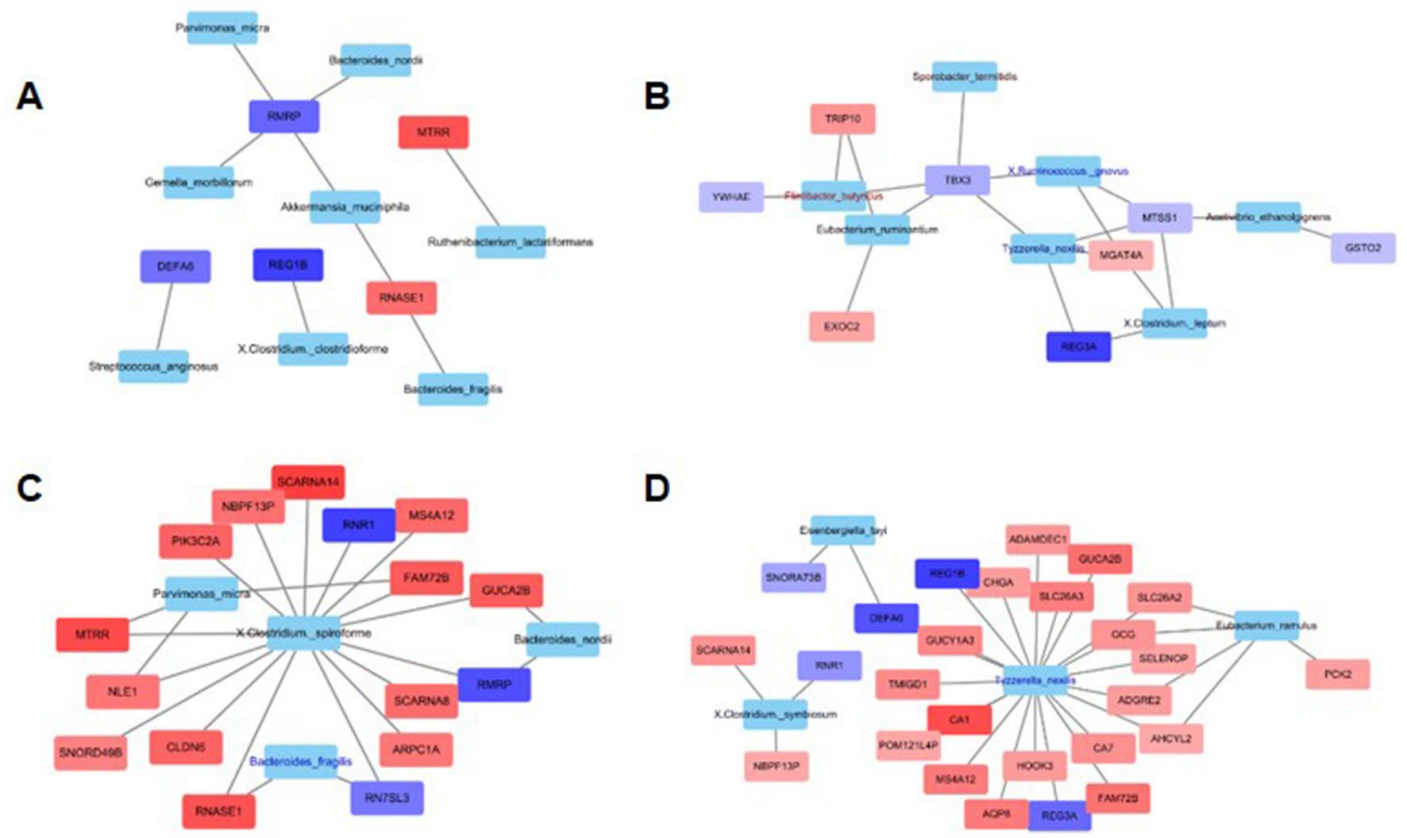
Interaction networks between differentially expressed genes (DEGs) and relative abundance of microbial strains. (**A**) Network analysis of 13 advanced colorectal cancer (ACRC) samples and 10 high-risk adenoma (HRA) samples versus 7 normal control (NC) samples. (**B**) Network analysis of 13 ACRC samples versus 10 HRA samples. (**C**) Network analysis of 13 ACRC samples versus 7 NC samples. (**D**) Network analysis of 10 HRA samples and 7 NC samples. Genes that are enriched in the word group are indicated in red; all other genes are in blue. Species with high relative abundance in the word group are indicated in red; all other species are in blue.

We then built a network to visualize the relationship between the bacteria and host gene expression during CRC initiation and progression. In the network analysis, we compared NC samples and disease samples (HRA + ACRC) (Fig. 4A), as well as the three original pairings (Fig. 4B–D). For genes, overexpression during CRC progression (NC to HRA to ACRC) is indicated in red, and downregulation is indicated in blue. For microbiome species, increased abundance during progression is indicated in red and decreased abundance in blue. Then, we provided the results of network analysis as four correlation plots (Fig. S2). We identified seven, four, and four species from network analysis in the ACRC-HRA, ACRC-NC, and HRA-NC pairings, respectively (Fig. S2A, S2B, and S2C), and 8, 16, and 26 genes, respectively. In the ACRC-HRA network (Fig. 4B), only eight genes were connected to seven species. Therefore, the correlation patterns of the eight genes were not detected. *C. spiroforme* was connected to 16 genes in the ACRC-NC network (Fig. 4C,D), and *T. nexilis* had 20 gene connections in the HRA-NC network. Most genes were positively correlated; *RNR1* and *RMRP* were negatively correlated in the ACRC-NC network (Fig. S2D), and *RNR1* was negatively correlated in the HRA-NC network (Fig. S2E). Therefore, our results suggest that *C. spiroforme* and *T. nexilis* are hub bacteria in the development and progression of CRC, and these bacteria can be candidates for the detection of CRC, including precancerous lesions.

### Analysis of correlation between omics data and sociomedical factors

The gut microbiome is a crucial environmental factor in the development of CRC, but its composition can also be affected by external factors such as dietary patterns and psychiatric factors^35^. In order to exclude such external interference and investigate the effect of external factors on metagenomic and RNA-seq results, sociomedical factors were also included in the integrated analysis. The 88 questionnaire-based sociomedical factors and the methods of analysis are summarized in Table S3. We obtained two correlation results between gene expression and sociomedical factors and between microbial distribution and sociomedical factors. The correlation coefficients were visualized as a heatmap, and clustering analysis was performed for each variable. From 88 survey questions, there were six clusters associated with gene expression and five clusters associated with microbial distribution were visualized as two heatmaps (Fig. S3 and Table S4). In both analyses, six clustered variables are listed in the heatmap. “Vomit” and “MtSor” were commonly clustered in both analyses, and positively and negatively correlated genes and microbes are listed. Psychiatric factors were closely located in the cluster. In the gene analysis, DEF (defensin alpha) and REG (regenerating family member) genes had higher correlation coefficients with diet. In the microbiome analysis, *E. fergusonii* showed higher correlation coefficients. However, the sociomedical factors did not affect the mucosal microbiome or gene expression during CRC initiation and progression.

## Discussion

Abnormal gene expression in the intestinal mucosa along with an imbalance in the intestinal microflora is one of the main causes of colorectal disease, and several mechanisms by which intestinal microbes and abnormal gene expression affect the development of colonic tumors have been suggested^27^. In this study, we integrated the results of RNA-seq, metagenomics, and sociomedical pattern analysis of ACRC, HRA, and NC samples to determine significant differences. We identified the diversity of the microbiome, showed a correlation between gene expression and the microbiome, and performed network analysis. We separated the signatures between the three groups and visualized distinct patterns. Our results provide a basis for manipulating the microbiome in treatment strategies for colorectal diseases.

Dysbiosis due to environmental factors such as dietary pattern or genetic variations can disrupt the immune system and may promote colorectal neoplasm^36–38^. The gut microbiota change can alter the efficacy of CRC treatment by increasing the sensitivity of chemotherapeutic agents, radiotherapy, and immune checkpoints inhibitors and reduced the toxicity of these treatment modalities^39^. Recent scientific evidence suggests that colorectal microbiota modification can inhibit ACRC progression and improve the treatment effect in ACRC^40^. A literature survey revealed that changing the colorectal microbiota composition by probiotics, prebiotics, and diet protects ACRC patients from treatment-associated adverse effects^18,40–42^. This study provides insights into the association between colorectal microbiota and colorectal diseases (including ACRC and HRA) to provide innovative strategies for enhancing the safety and efficacy of ACRC and HRA therapy.

Many studies have examined specific gut bacterial species associated with colorectal diseases. A typical example is sulfidogenic bacteria. Hydrogen sulfide-producing bacteria such as *Fusobacterium*, *Desulfovibrio*, and *B. wadsworthia* are known to be involved in ACRC development through the production of DNA-damaging hydrogen sulfide^43–45^. In addition, patients with beneficial gut microbiota, such as *B. longum*, *Ruminococcaceae* spp., *E. faecium*, *Faecalibacterium* spp., and *C. aerofaciens*, have superior systemic and antitumor immunity compared to patients with low strain diversity and relatively high abundance of *Bacteroidetes*^41,46^. This phenomenon suggests that intestinal microbiota can modulate immune function in the intestine and increase tumor immunity.

Most existing studies on gut microbiota in CRC have analyzed gut microbiota in feces. Examination of the feces is non-invasive and may be appropriate as a screening test, but there may be variables that affect the metagenomic results, such as the collection process, dietary pattern, and antibiotic administration^10,12^. In this study, the microbiome genome was profiled in the colonic mucosa using samples removed during colonoscopy. We were able to identify species with high relative abundance in ACRC- and HRA-derived mucosa (three *Bacteroides* and two *Clostridium* species) and extracted genes that were highly correlated with these bacterial species.

Understanding the molecular mechanisms of CRC development and progression is key to early diagnosis and the development of personalized medicines. Several previous studies have clarified the importance of the interaction between host cells and the microbiome in the pathogenesis of CRC^24,31,47,48^. To understand the role of these interactions in the adenoma-carcinoma sequence, we correlated host gene expression and mucosal microbiome genomic composition data using microbiota and RNA-seq data in HRA, ACRC, and NC samples. In the correlation and network analyses of mucosal-derived microorganisms and gene expression, *T. nexilis* was a hub species related to DEGs in HRA and NC samples and in ACRC and HRA samples. In addition, *C. spiroforme* was identified as a hub species related to differential gene expression when comparing ACRC and NC samples. *C. spiroforme* had a strong positive relationship with *NBPF13P* and a strong negative correlation with *RMRP*.

*REG3A* was found to be elevated in ACRC samples compared to NC samples. High *REG3A* levels are correlated with larger tumor size, poorer tumor differentiation, higher tumor stage, and lower survival rate^49^. *REG3A* has been shown to have pro-tumorigenic effects, including promotion of cell proliferation, inhibition of cell apoptosis, and regulation of cancer cell migration by activating AKT and ERK1/2 pathways in gastric cancer cells^50^. *REG3A* has also been considered to play a key role in inflammation-linked pancreatic carcinogenesis^51,52^. Therefore, *REG3A* may serve as a promising therapeutic target in ACRC. We are the first to identify a relationship between ACRC and *NBPF13P*. We revealed a relationship between the microbiome and *NBPF13P*, which could provide a new pathway for targeting in colorectal diseases.

The colon has the highest load of gut microbiota, with over 10^11^ bacteria per milliliter. Colonic symbionts can be classified according to their anatomical distribution as (1) luminal-resident bacteria, (2) mucous-resident bacteria, (3) epithelial-resident bacteria, and (4) lymphoid tissue-resident symbionts. Intestinal epithelial cells (IECs) play an important role in innate immunity by forming a physical barrier against environmental stimuli, including gut genetic toxins, and maintaining a balance between commensal bacteria and host cells^53,54^. Although this barrier is sterile, invasive bacteria, including adherent-invasive *Escherichia coli*, segmented filamentous bacilli, *Enterococcus faecalis, Bacteroides fragilis*, and *Clostridium* spp., can reside in and attach to IECs^55,56^. This can lead to chronic inflammation of the mucous membrane, which is one of the critical pathogeneses of inflammatory bowel disease and CRC and correlates with disease severity. Since our study analyzed the microbiota of the colonic mucosa, it is difficult to exclude the possibility that a large portion of the microbiota present in IECs might be included in the metagenomic analysis. *Tyzzerella* and *Clostridium*, which are correlated with CRC progression and differential gene expression, are known to reside in IECs.

*Clostridium* spp., a representative epithelium-resident bacteria, forms endospores and has strong dissemination power, survival, and resistance to antibiotics^57^. Spore-forming bacteria have the following characteristics: resistance to antibiotic treatment, strong binding properties, high permeability, and harmful spores^58^. The role of sporobiota in the pathogenesis and progression of CRC remains unclear and needs to be elucidated. Our results suggest that gut sporobiota may be important in the pathogenesis of CRC. Understanding the mechanism of CRC pathogenesis is useful not only for the development of targeted therapeutics, which could potentially define markers and guide precision medicine, but also for the early detection and prevention of CRC. Identification of the exact role of sporobiota in colorectal tumorigenesis will help us understand the current limitations of gut microbiota modulations, such as antibiotic administration, diet modification, and probiotic administration, for CRC prevention and treatment and can suggest new target therapies for CRC.

In this study, we also investigated the association between the composition of the intestinal microflora and dietary patterns and other environmental factors. No definitive difference was observed in the gut mucosal microbiota diversity between HRA, ACRC, and NC samples. This result is not in line with those of previous studies using fecal microbiota analysis. This suggests that environmental factors, including dietary patterns and socioeconomic, psychiatric, and clinical factors may have less influence on the gut mucosal microbiota diversity compared to that of feces. Future large-scale studies are needed to clarify this.

Our study had several limitations. First, whether mucosal microbiota analysis reflects the effect of microbiota on the development and progression of CRC may be controversial. Since our colonic tissue was obtained during colonoscopy, it is possible that the results of the metagenomic analysis may have been affected by the bowel preparation process. Second, because of the small number of samples, differences in the mucosal microbiome and gene expression according to clinical characteristics of ACRC and HRA, such as tumor stage, were not fully analyzed. Third, we used 16S rRNA in mucosal microbiome analysis. 16S rRNA analysis has a limitation in that the accuracy of taxonomic resolution to species is lower compared to that of full-length sequencing (shotgun metagenome). Although similar patterns were detected between two methods^59^, relatively low resolutions with biases and errors were predicted in 16S rRNA analysis for taxonomic classification (78% of species-level vs 98% genera-level)^60^. The taxonomic assignment of species is more important and crucial than that of genus level. So, additional studies, including new omics techniques and culturomics, should be performed to confirm and validate our results.

## Conclusions

In summary, we provided a set of candidate correlations and interactions between the gut microbiota and host genes in ACRC and HRA samples that are distinct from those of NC samples. This demonstrates the correlation between the microbiome and gene expression in the colonic mucosa during disease progression from NC to HRA to ACRC. Our results may provide clinicians and researchers with a basis for diagnosis and targeted treatment using gut mucosal microbiota, suggesting the relevance of sporobiota in CRC progression.

## Methods

### Study design and participants

This study was approved by the Institutional Review Board of Korea University Guro Hospital (2019GR0341). Colonic mucosal tissues were obtained from colonoscopies after bowel cleansing with 2L-based PEG (polyethylene glycol)-based laxatives at the Korea University Guro Hospital. None of the enrolled patients had an acute infection within the 3 months before the procedure. ACRC and advanced HRA tissues were obtained from the core lesion, as depicted in Fig. S1A. NC samples were obtained from the sigmoid colons of patients with normal colonoscopy findings who underwent routine colonoscopic CRC screening. Two pinch biopsies (~ 3 × 3 mm) from the lesions or sigmoid colons were obtained using colonoscopic biopsy forceps, one for RNA-seq and one for metagenomic sequencing. All tissues were placed into RNA stabilization solution (Thermo Fisher Scientific, Waltham, MA, USA) and stored for 24 h at 4 °C prior to freezing at − 80 °C to prevent anaerobic bacteria from being exposed to oxygen and to avoid bacterial overgrowth before DNA extraction. RNA-seq and 16S metagenomics sequencing were performed on 13 ACRC samples, 10 HRA samples, and 7 NC samples. The demographic and basal characteristics of the enrolled patients are summarized in Fig. S1B.

### Assessment of sociomedical factors

We considered social lifestyle factors, family history of cancer using a family tree, medical histories, gastrointestinal symptoms, including the Bristol stool form scale, and psychosocial factors using CES. Diet patterns were assessed using the Korean standard nutrition questionnaire (Fourth version; 2007–2009), which is a 100-item questionnaire. Detailed information on the questionnaire is provided in Table S3.

### DNA extraction and 16S rRNA sequencing

DNA was extracted from the colonic mucosa samples using the DNeasy Blood and Tissue kit (Qiagen, Germany). The bacterial V3–V4 region of 16S rRNA gene was used for PCR amplification. The primers used were 338F (5′-ACTCCTACGGGAGGCAGCA-3′) and 806R (5′-GGACTACHVGGGTWTCTAAT-3′). The PCR process was initial denaturation at 95 °C for 5 min, 28 cycles consisting of 15 s denaturation at 95 °C, 30 s annealing at 55 °C and 30 s extension at 72 °C, with a final extension at 72 °C for 10 min. Amplicons of the V3–V4 region were maintained in equal amounts, and pair-end 2 × 300 bp was sequenced by the Illumina MiSeq platform with the MiSeq Reagent Kit v3. The raw pair-ended amplicon sequence reads were retrieved.

### 16S rRNA analysis and diversity analysis

We processed the FASTQ files using FastQC^61^ to perform quality control of the raw sequences. The raw 16S amplicon sequences were processed by QIIME2 v1.8.0 with default parameters. We then used SHI7^62^ for trimming Nextera adapters and stitching paired-end reads and performed quality trimming at both ends of the stitched reads until a minimum Phred score of 32 was reached. These merged and filtered reads were used for closed-reference operational taxonomic unit (OTU) picking, and the OTUs were determined by de novo clustering of the sequences with a 97% sequence identity cut-off by QIIME. We performed alpha- and beta-diversity analyses in R using the vegan^63^ and phyloseq^64^ packages. Based on the OTU table, we calculated the average richness estimate for each alpha-diversity metric (Chao1, observed OTUs, and Shannon) (Table S5).

### Bioinformatics and visualization

We used the RNA-seq data from our previous study^32^. From 46,851 features, 763 were selected using the “anova” function in R (*p* < 1 × 10^–6^). The final dataset included 529 species from metagenomics (Table S6), 763 genes from RNA-seq, and 93 variables from the survey results from the 30 samples (ACRC, n = 13, HRA, n = 10, and NC, n = 7). A total of 1385 features were normalized to values between 0 and 1. Principal component analysis (PCA) was performed by the “prcomp” function in R. To display the ternary plot, we used the “triax.plot” function of the “plotrix” package in R.

### Integrated analysis of interaction between microbiome and host gene expression

The normalized 1385 features were used for the correlation analysis and visualized by correlation plots and heatmaps. The correlation analysis were performed by “cor.test” default function in R, and visualized by “corrplot” function of the “corrplot” package in R. Scatter plots were visualized by “ggplot” function of the “ggplot2” package in R. Network analysis was visualized by using Cytoscape, and each features was used as color keys.

### Ethics approval and consent to participate

All cases were over 18 and informed consent was obtained in all cases. All methods were carried out in accordance with relevant guidelines and regulations (Declaration of Helsinki). This study was approved by the Institutional Review Board at Korea University Guro Hospital (2019GR0341).

## Supplementary Information

Supplementary Information.

Supplementary Table S4.

Supplementary Table S6.

## Data Availability

The datasets generated during the current study are not publicly available due to Personal Information Protection Act of Republic of Korea and IRB recommendation of Korea University Guro Hospital but are available from the corresponding author on reasonable request.

## Abbreviations

ACRC: Advanced colorectal cancer
ANOVA: Analysis of variance
AUC: Area under the curve
CRC: Colorectal cancer
DEG: Differentially expressed gene
HRA: High-risk adenoma
IEC: Intestinal epithelial cell
NBPF13P: Neuroblastoma breakpoint family member 13, pseudogene
NC: Normal control
OTU: Operational taxonomic unit
PC: Principal component
PCA: Principal component analysis
PV: P-value
REG3A: Regenerating family member 3 alpha
RMRP: RNA component of mitochondrial RNA processing endoribonuclease
RNA-seq: RNA sequencing
RNR1: RNA, ribosomal 45S cluster 1
FC: Fold change
