# Supplementary material for: Crosstalk between mucosal microbiota, host gene expression, and sociomedical factors in the progression of colorectal cancer
Source: Sci Rep. 2022 Aug 4;12:13447. doi: 10.1038/s41598-022-17823-7 (PMC9352898; doi:10.1038/s41598-022-17823-7)
Supplement: Supplementary file 1 — Supplementary Information. [file 41598_2022_17823_MOESM1_ESM.docx]

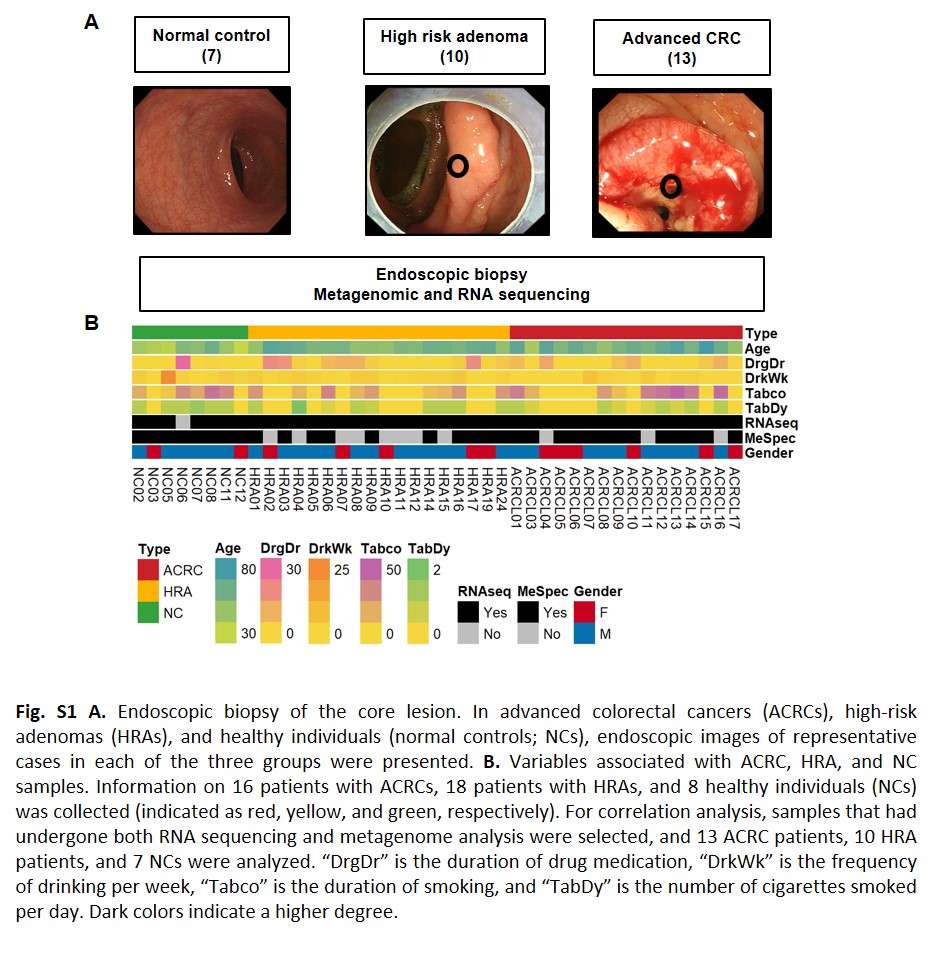


**Fig. S1** **Study design and basal characteristics of enrolled patients**

**A.** Endoscopic biopsy of the core lesion. In advanced colorectal cancers (ACRCs), high-risk adenomas (HRAs), and healthy individuals (normal controls; NCs), endoscopic images of representative cases in each of the three groups were presented. **B.** Variables associated with ACRC, HRA, and NC samples. Information on 16 patients with ACRCs, 18 patients with HRAs, and 8 healthy individuals (NCs) was collected (indicated as red, yellow, and green, respectively). For correlation analysis, samples that had undergone both RNA sequencing and metagenome analysis were selected, and 13 ACRC patients, 10 HRA patients, and 7 NCs were analyzed. “DrgDr” is the duration of drug medication, “DrkWk” is the frequency of drinking per week, “Tabco” is the duration of smoking, and “TabDy” is the number of cigarettes smoked per day. Dark colors indicate a higher degree.


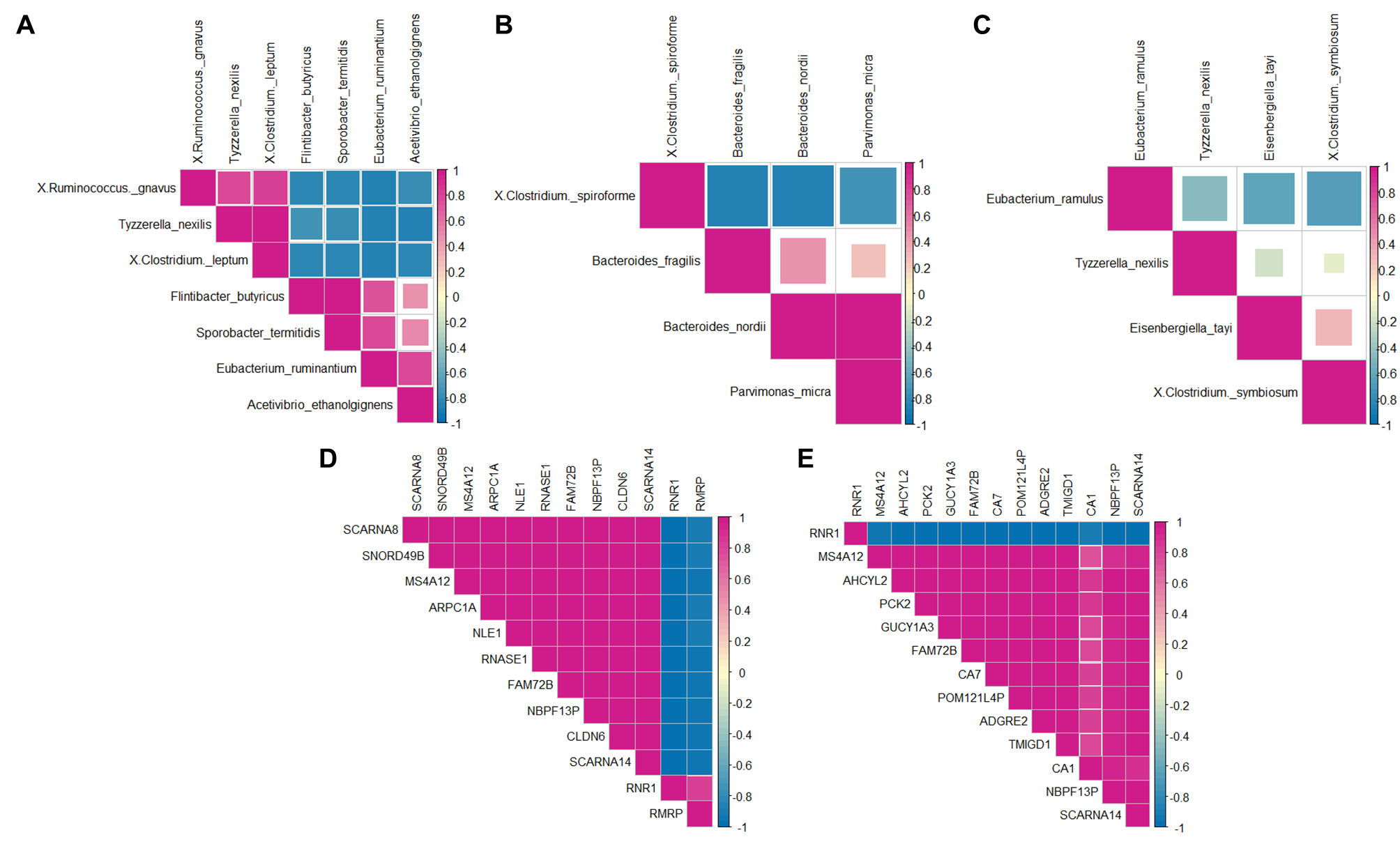


**Fig. S2 Correlation plot between two conditions from the metagenome and RNA-seq dataset.** **A.** Correlations between 7 species were analyzed from 13 advanced colorectal cancers (ACRCs) and 10 high-risk adenomas (HRAs). **B.** Correlations between 4 species were analyzed from 13 ACRCs and 7 normal controls (NCs). **C.** Correlations between 4 species were analyzed from 10 HRAs and 7 NCs. **D.** Correlations between 12 genes were analyzed from 10 HRAs and 7 NCs. **E.** Correlations between 13 genes were analyzed from 13 ACRCs and 7 NCs.

**
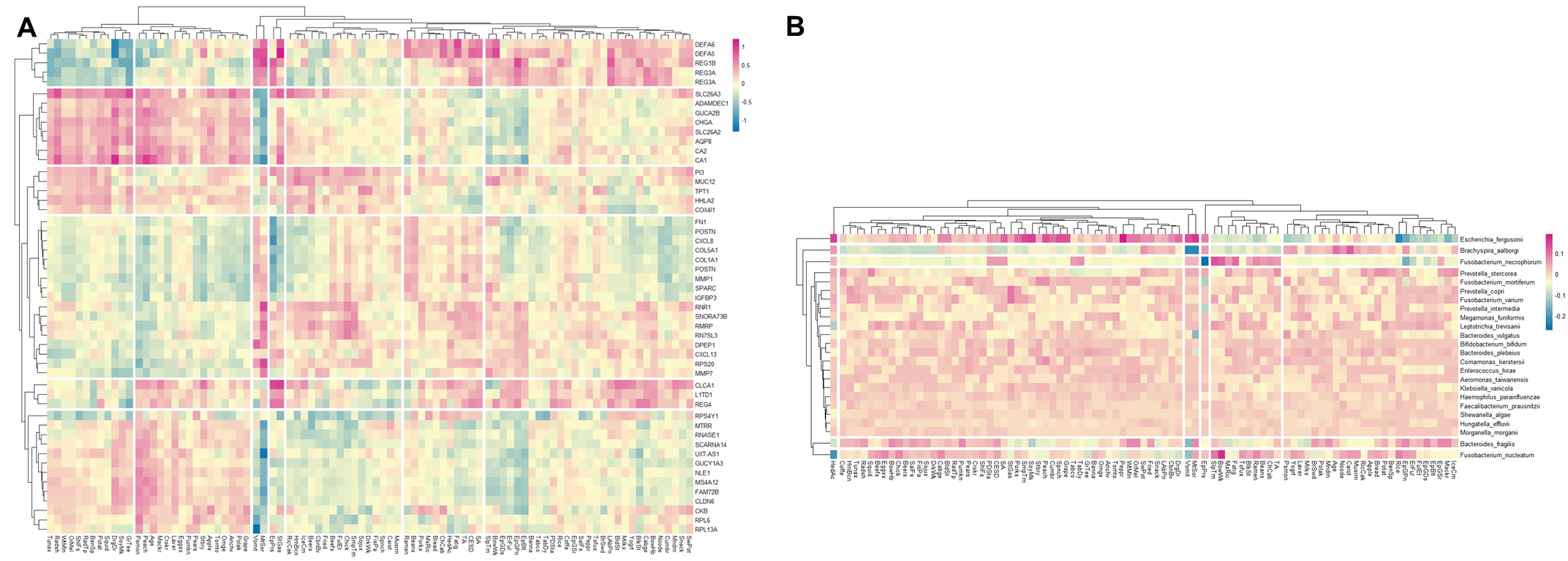
**

**Fig. S3** Heatmap of correlation between lifestyle patterns and omics data. Eighty-eight continuous variables were selected from the survey and compared with RNA sequencing and metagenome results. **A.** Correlations between 88 survey questions and 51 genes from RNA-seq analysis. **B.** Correlations between 88 survey questions and 24 species from metagenome analysis. Raw numeric matrix of **Fig. S3** heatmaps were provided as separated **Table S4**.

**Table S1** List of enrolled patients

| Case | Age | Gender | Case | Age | Gender | Case | Age | Gender |
| --- | --- | --- | --- | --- | --- | --- | --- | --- |
| HRA01 | 48 | Male | ACRC01 | 75 | Male | NC02 | 42 | Male |
| HRA02 | 78 | Female | ACRC03 | 39 | Male | NC03 | 34 | Female |
| HRA03 | 74 | Male | ACRC04 | 77 | Female | NC05 | 29 | Male |
| HRA04 | 71 | Male | ACRC05 | 59 | Female | NC06 | 57 | Male |
| HRA05 | 60 | Male | ACRC06 | 72 | Female | NC07 | 53 | Male |
| HRA06 | 62 | Male | ACRC07 | 63 | Male | NC08 | 67 | Male |
| HRA07 | 64 | Female | ACRC08 | 48 | Male | NC11 | 45 | Male |
| HRA08 | 50 | Male | ACRC09 | 62 | Male | NC12 | 25 | Female |
| HRA09 | 51 | Male | ACRC10 | 70 | Female | Average | 44.0(±14.4) | M: 6 F: 2 |
| HRA10 | 49 | Female | ACRC11 | 61 | Male |  |  |  |
| HRA11 | 59 | Male | ACRC12 | 73 | Male |  |  |  |
| HRA12 | 57 | Male | ACRC13 | 81 | Male |  |  |  |
| HRA13 | 68 | Male | ACRC14 | 63 | Male |  |  |  |
| HRA14 | 66 | Male | ACRC15 | 89 | Female |  |  |  |
| HRA15 | 62 | Male | ACRC16 | 68 | Male |  |  |  |
| HRA16 | 70 | Female | ACRC17 | 48 | Female |  |  |  |
| HRA19 | 73 | Female | Average | 65.5(±13.0) | M: 10 F: 6 |  |  |  |
| HRA24 | 50 | Male |  |  |  |  |  |  |
| Average | 61.8(±9.5) | M: 13 F: 5 |  |  |  |  |  |  |

**Table S2** Raw numeric matrix of **Fig. 2C** heatmap. The heatmap provides the relative abundance of significantly different between three groups. Eight significantly different in three groups were discovered through ANOVA (p-value < 0.05)

| **Taxon name** | ***Bacteroides***  ***coprocola*** | ***Bacteroides***  ***plebeius*** | ***Bacteroides***  ***vulgatus*** | ***Dorea***  ***formicigenerans*** | ***Lachnoclostridium***  ***pacaense*** | ***X.Clostridium.***  ***scindens*** | ***X.Clostridium.***  ***spiroforme*** | ***Aquabacterium***  ***parvum*** |
| --- | --- | --- | --- | --- | --- | --- | --- | --- |
| HRA01 | 0 | 0 | 0.000224 | 0 | 0 | 0 | 0 | 0 |
| HRA03 | 0 | 0.004409 | 0.024497 | 0 | 0 | 0.005847 | 0.005945 | 0 |
| HRA05 | 0 | 0.011106 | 0.026151 | 0 | 0.002763 | 0 | 0.003583 | 0 |
| HRA06 | 0 | 0 | 0.004122 | 0 | 0 | 0 | 0 | 0 |
| HRA09 | 0 | 0.004354 | 0.000323 | 0 | 0 | 0 | 0 | 0 |
| HRA14 | 0.006797 | 0.00189 | 0.002913 | 0 | 0.001129 | 0 | 0 | 0 |
| HRA16 | 0.012498 | 0.030763 | 0.10216 | 0.006762 | 0.004647 | 0 | 0.006762 | 0 |
| HRA17 | 0.001512 | 0.003482 | 0.087302 | 0.013648 | 0 | 0 | 0.028632 | 0 |
| HRA19 | 0 | 0.249768 | 0 | 0.010393 | 0.002324 | 0.005578 | 0.000232 | 0.002358 |
| HRA20 | 0.01516 | 0.004677 | 0.009237 | 0.00682 | 0.004989 | 0 | 0.001208 | 0.002494 |
| HRA22 | 0 | 0.007514 | 0.005312 | 0.004146 | 0.002073 | 0.002591 | 0.002634 | 0.003066 |
| HRA24 | 0.00057 | 0.00054 | 0 | 0 | 3.00E-05 | 0 | 0.00093 | 0.00066 |
| ACRCL01 | 0 | 0 | 0.080907 | 0.006621 | 0.006002 | 0 | 0 | 0 |
| ACRCL02 | 0 | 3.52E-05 | 0 | 0 | 0 | 0 | 0 | 0 |
| ACRCL03 | 0 | 0 | 3.15E-05 | 0 | 0.000252 | 0 | 0 | 0 |
| ACRCL05 | 0 | 0.010952 | 0.003526 | 0.002272 | 0.003391 | 0 | 0 | 0 |
| ACRCL06 | 0 | 0 | 0.01669 | 0.000847 | 0.002933 | 0 | 0 | 0 |
| ACRCL07 | 0 | 0 | 0.003276 | 0 | 0 | 0 | 0.004302 | 0 |
| ACRCL08 | 0 | 0 | 0 | 0 | 0.000472 | 0.000157 | 0 | 0 |
| ACRCL09 | 0.008104 | 0.039915 | 0.022492 | 0.000698 | 0 | 0 | 0.006101 | 0 |
| ACRCL10 | 0.001269 | 0.000968 | 0.00384 | 0 | 0 | 0.001169 | 0 | 0 |
| ACRCL12 | 0.005913 | 0.07337 | 0.075322 | 0.000919 | 0.000603 | 0 | 0.000459 | 0 |
| ACRCL13 | 0 | 0 | 0.011417 | 0 | 0 | 0 | 0 | 0 |
| ACRCL14 | 0 | 0.000277 | 0.001445 | 0.000184 | 0.000307 | 0 | 0.000307 | 0 |
| ACRCL15 | 0 | 0 | 0.000683 | 0 | 0 | 0 | 2.36E-05 | 0 |
| ACRCL17 | 0 | 0 | 0 | 0 | 0 | 0 | 0 | 0 |
| NC02 | 0 | 0.017179 | 0.041543 | 0 | 0.001171 | 0 | 0.008564 | 0 |
| NC03 | 0.064949 | 0.182552 | 0.237307 | 2.42E-05 | 0.006521 | 0 | 0.01256 | 0 |
| NC05 | 0 | 0 | 0.110348 | 0.00187 | 0 | 0 | 0.018271 | 0 |
| NC06 | 0 | 0.197691 | 0.045423 | 0.00147 | 0.014183 | 0 | 0.019025 | 0 |
| NC07 | 0 | 0 | 0.000623 | 0 | 0 | 0 | 0 | 0 |
| NC08 | 0 | 0.004519 | 0.097183 | 0 | 0 | 0 | 0.008536 | 0 |
| NC09 | 0 | 0.175228 | 0.249292 | 0.000799 | 0.006418 | 0 | 0.002906 | 0 |
| NC10 | 0.115134 | 0.0601 | 0.307051 | 0.001019 | 0.00424 | 0 | 0.000385 | 0 |
| NC11 | 0.086021 | 0.071233 | 0.00741 | 0 | 0.002964 | 0 | 0.020974 | 0 |
| NC12 | 0 | 0.004417 | 0.076317 | 0.001613 | 0 | 0 | 0 | 0 |
| NC13 | 0.011602 | 0.005659 | 0.005546 | 0 | 0.000764 | 0 | 0.007753 | 0 |
| NC14 | 0.09232 | 0.18987 | 0.041263 | 0.000116 | 0.011653 | 0 | 0.001424 | 0 |
| NC15 | 0.012868 | 0.078887 | 0.033147 | 0.000995 | 0.006039 | 0 | 0 | 0 |
| PV | 0.010604 | 0.024834 | 0.004378 | 0.033381 | 0.027696 | 0.04796 | 0.029685 | 0.012767 |

**Table S3** List of questionnaire items

| **Category** | **Items** | **Abbreviation** | **Degree** |
| --- | --- | --- | --- |
| **Demographics** | Gender | Gender | male vs. female |
|  | Age | Age | Continuous variable |
|  | Smoking status | Tabco | never smoker vs. other |
|  | Place of birth | HmTwn | Urban vs country |
|  | Family History |  | Negative family history vs immediate family history vs immediate family history) |
|  | Family History of CRC |  | Negative family history vs immediate family history vs immediate family history) |
|  | Hobby | Hobby | (indoor vs outdoor activities) |
|  | Smart phone | SmpTm | Hours /day |
|  | Pet animal | PetOX | none vs type |
|  | medication history | PreDs | Hypertension-A, Diabetes-B, Respiratory-C, Digestive-D, Other-E, Missing-F |
| **Psychological assessments** | CES-D | CESD | scoring (normal, mild, moderate, severe) |
|  | State-anxiety | SA | scoring (normal, mild, moderate, severe) |
|  | Trait-anxiety | TA | scoring (normal, mild, moderate, severe) |
|  | Sleep disturbance | SlpDt | sleeping pill vs none |
| **Bowel habit** | Frequency | BowWk | times/week |
|  | Bristol stool form scale | Bristol | 1. to 7 |
| **Dietary pattern** | Rice | Rice | rarely, often, usually, always |
|  | mixed rice | MxRic | rarely, often, usually, always |
|  | Ramen | Ramen | rarely, often, usually, always |
|  | Noodle | Noode | rarely, often, usually, always |
|  | Bread | Bread | rarely, often, usually, always |
|  | Snack | RicCak | rarely, often, usually, always |
|  | Tofu | Tofux | rarely, often, usually, always |
|  | Bean | Beanx | rarely, often, usually, always |
|  | Soybean milk | SoyMk | rarely, often, usually, always |
|  | Potato | Potat | rarely, often, usually, always |
|  | Sweet potato | SwPot | rarely, often, usually, always |
|  | Beef | Beefx | rarely, often, usually, always |
|  | Chicken | Chick | rarely, often, usually, always |
|  | Pork | Porkx | rarely, often, usually, always |
|  | Ham, Bacon | HmBcn | rarely, often, usually, always |
|  | Egg | Eggxx | rarely, often, usually, always |
|  | Mackerel | Mackr | rarely, often, usually, always |
|  | Tuna | Tunax | rarely, often, usually, always |
|  | Croaker | Crokr | rarely, often, usually, always |
|  | Pollack | Polak | rarely, often, usually, always |
|  | Anchovy | Anchv | rarely, often, usually, always |
|  | Squid | Squid | rarely, often, usually, always |
|  | Clam | ShlFs | rarely, often, usually, always |
|  | Salted fish | SalFs | rarely, often, usually, always |
|  | Chinese cabbage | ChCab | rarely, often, usually, always |
|  | White radish | Radsh | rarely, often, usually, always |
|  | Radish tops | RadTp | rarely, often, usually, always |
|  | Bean sprouts | BenSp | rarely, often, usually, always |
|  | Spinach | Spnch | rarely, often, usually, always |
|  | Cucumber | Cumbr | rarely, often, usually, always |
|  | Pepper | Peppr | rarely, often, usually, always |
|  | Carrot | Carot | rarely, often, usually, always |
|  | Pumpkin | Pumkn | rarely, often, usually, always |
|  | Cabbage | Cabge | rarely, often, usually, always |
|  | Tomato | Tomto | rarely, often, usually, always |
|  | Mushroom | Musrm | rarely, often, usually, always |
|  | Seaweed | BrSwd | rarely, often, usually, always |
|  | Dried seaweed | Laver | rarely, often, usually, always |
|  | Mandarin | Mndrn | rarely, often, usually, always |
|  | Persimmon | Psmon | rarely, often, usually, always |
|  | pear | Pears | rarely, often, usually, always |
|  | watermelon | WtMln | rarely, often, usually, always |
|  | oriental melon | OrMel | rarely, often, usually, always |
|  | strawberry | Stbry | rarely, often, usually, always |
|  | Grape | Grape | rarely, often, usually, always |
|  | Peach | Peach | rarely, often, usually, always |
|  | Apple | Apple | rarely, often, usually, always |
|  | Banana | Banna | rarely, often, usually, always |
|  | Orange | Ornge | rarely, often, usually, always |
|  | Milk | Milkx | rarely, often, usually, always |
|  | Yogurt | Yogrt | rarely, often, usually, always |
|  | Ice cream | IceCm | rarely, often, usually, always |
|  | Carbonated drink | CbnBv | rarely, often, usually, always |
|  | Coffee | Coffe | rarely, often, usually, always |
|  | Green tea | GrTee | rarely, often, usually, always |
|  | Hamburger | Hmbgr | rarely, often, usually, always |
|  | Pizza | Pizza | rarely, often, usually, always |
|  | Fried | Fried | rarely, often, usually, always |
| **GI symptom** | Epigastric pain | EpGPn | none vs mild vs moderate vs severe |
|  | Epigastric Discomfort | EpGDs | none vs mild vs moderate vs severe |
|  | Epigastric soreness | EpGSr | none vs mild vs moderate vs severe |
|  | Fullness | FulEt | none vs mild vs moderate vs severe |
|  | Early satiety | ErFul | none vs mild vs moderate vs severe |
|  | Nausea/Vomiting | Vomit | none vs mild vs moderate vs severe |
|  | Headache | HedAc | none vs mild vs moderate vs severe |
|  | Lower abdominal pain | LAbPn | none vs mild vs moderate vs severe |
|  | Flatulence | StGas | none vs mild vs moderate vs severe |
|  | Melena | BlkSt | none vs others |
|  | Hematochezia | BldSt | none vs others |
|  | Bowel habit change | BowHb | none vs others |
|  | Fatigue | Fatig | none vs mild vs moderate vs severe |

**Table S4** Raw numeric matrix of **Fig. S3** heatmaps. The heatmap means correlation between lifestyle patterns and omics data. First and second tab were fold change and p-value of RNA-seq results, and third and fourth tab were fold change and p-value of metagenome results.

**Table S5** Sequencing results, community richness, and diversity of metagenome analysis

| **SampleName** | **Total Bases** | **Read Count** | **N (%)** | **GC (%)** | **Q20 (%)** | **Q30 (%)** | **OTUs** | **Chao1** | **Shannon** | **Inverse Simpson** | **Good's Coverage** |
| --- | --- | --- | --- | --- | --- | --- | --- | --- | --- | --- | --- |
| HRA01 | 39,847,579 | 86,724 | 0 | 54.31 | 97.34 | 89.59 | 53 | 53.00 | 2.6163 | 0.6280 | 1.0000 |
| HRA03 | 34,330,938 | 75,777 | 0 | 53.18 | 97.46 | 89.63 | 106 | 106.50 | 4.9027 | 0.9198 | 0.9999 |
| HRA05 | 46,275,112 | 100,421 | 0 | 53.68 | 97.51 | 90.01 | 72 | 72.00 | 2.4589 | 0.5193 | 1.0000 |
| HRA06 | 36,945,834 | 82,855 | 0 | 49.6 | 97.64 | 89.95 | 45 | 45.50 | 2.7018 | 0.6829 | 0.9999 |
| HRA09 | 48,659,895 | 107,307 | 0 | 48.18 | 97.84 | 90.69 | 40 | 40.00 | 2.4628 | 0.7076 | 1.0000 |
| HRA14 | 42,252,323 | 93,531 | 0 | 51.2 | 97.62 | 90.11 | 101 | 102.50 | 3.7473 | 0.7974 | 0.9999 |
| HRA16 | 37,630,252 | 82,795 | 0 | 51.6 | 97.59 | 90.02 | 78 | 81.00 | 4.7895 | 0.9405 | 0.9999 |
| HRA17 | 54,469,323 | 121,518 | 0 | 52.84 | 97.5 | 89.9 | 206 | 209.50 | 5.1194 | 0.9326 | 0.9997 |
| HRA19 | 36,427,933 | 81,030 | 0 | 53.32 | 97.46 | 89.58 | 90 | 90.00 | 4.1287 | 0.8763 | 1.0000 |
| HRA24 | 44,955,516 | 101,917 | 0 | 49.59 | 97.5 | 89.7 | 66 | 66.00 | 0.9428 | 0.2036 | 1.0000 |
| ACRC01 | 41,066,631 | 91,078 | 0 | 51.63 | 97.56 | 89.85 | 68 | 68.00 | 4.3870 | 0.9179 | 1.0000 |
| ACRC03 | 42,182,441 | 93,322 | 0 | 50.34 | 97.45 | 89.79 | 35 | 35.50 | 2.1179 | 0.6934 | 0.9999 |
| ACRC05 | 35,238,467 | 77,447 | 0 | 52.3 | 97.26 | 89.29 | 91 | 94.33 | 4.0240 | 0.8302 | 0.9998 |
| ACRC06 | 43,195,502 | 93,106 | 0 | 54.77 | 97.27 | 89.5 | 39 | 39.00 | 1.9396 | 0.5606 | 1.0000 |
| ACRC07 | 39,872,150 | 89,554 | 0 | 50.78 | 97.82 | 90.4 | 54 | 54.00 | 1.5525 | 0.3370 | 1.0000 |
| ACRC08 | 47,664,434 | 105,328 | 0 | 52.11 | 97.46 | 89.87 | 94 | 99.08 | 2.2174 | 0.6384 | 0.9996 |
| ACRC09 | 40,287,282 | 89,673 | 0 | 52.25 | 97.5 | 89.81 | 136 | 136.00 | 6.0515 | 0.9772 | 1.0000 |
| ACRC10 | 40,059,863 | 88,202 | 0 | 53.7 | 97.39 | 89.54 | 128 | 128.33 | 4.5545 | 0.8514 | 0.9999 |
| ACRC12 | 39,784,732 | 88,129 | 0 | 51.84 | 97.57 | 89.91 | 149 | 151.00 | 5.5225 | 0.9598 | 0.9999 |
| ACRC13 | 43,944,824 | 94,640 | 0 | 54.24 | 97.42 | 89.83 | 29 | 29.00 | 1.7849 | 0.5577 | 1.0000 |
| ACRC14 | 49,183,518 | 108,580 | 0 | 49.89 | 97.71 | 90.28 | 91 | 95.23 | 3.3345 | 0.8171 | 0.9997 |
| ACRC15 | 44,911,151 | 98,342 | 0 | 50.28 | 97.58 | 90.16 | 66 | 66.33 | 2.8501 | 0.7563 | 1.0000 |
| ACRC17 | 37,641,851 | 84,348 | 0 | 50.67 | 97.64 | 89.88 | 41 | 41.00 | 2.4420 | 0.7180 | 1.0000 |
| NC02 | 43,066,761 | 94,921 | 0 | 52.36 | 97.35 | 89.49 | 118 | 118.00 | 5.0587 | 0.9434 | 1.0000 |
| NC03 | 40,782,476 | 89,757 | 0 | 50.73 | 97.63 | 90.28 | 115 | 115.50 | 4.4689 | 0.8950 | 1.0000 |
| NC05 | 46,919,765 | 105,692 | 0 | 49.77 | 97.5 | 89.69 | 67 | 67.00 | 3.0567 | 0.6973 | 1.0000 |
| NC06 | 49,054,276 | 107,975 | 0 | 51.75 | 97.57 | 90 | 102 | 102.00 | 4.7833 | 0.9289 | 1.0000 |
| NC07 | 41,965,056 | 91,219 | 0 | 54.26 | 97.29 | 89.41 | 36 | 36.00 | 1.9623 | 0.5410 | 1.0000 |
| NC08 | 46,349,146 | 101,091 | 0 | 52.72 | 97.37 | 89.6 | 73 | 73.00 | 3.9013 | 0.8261 | 1.0000 |
| NC11 | 36,500,682 | 79,913 | 0 | 52.58 | 96.5 | 86.67 | 61 | 61.00 | 3.2255 | 0.7759 | 1.0000 |
| NC12 | 39,352,490 | 87,250 | 0 | 51.01 | 97.63 | 90.05 | 45 | 45.00 | 3.1020 | 0.8051 | 1.0000 |

**Table S6** Taxonomy abundance counts (provided as separated excel file)
